# Measuring the barriers against seeking consultation for urinary incontinence among Middle Eastern women

**DOI:** 10.1186/1472-6874-10-3

**Published:** 2010-01-27

**Authors:** Ahmed S El-Azab, Omar M Shaaban

**Affiliations:** 1 Department of Urology, Section of Female Urology and Neurourology; Assiut University Hospital, Assiut University, Assiut, Egypt; 2 Woman's Health Centre, Department of Obstetrics and Gynaecology; Assiut University Hospital, Assiut University, Assiut, Egypt

## Abstract

**Background:**

Existing questionnaires to assess barriers against consultation for urinary incontinence (UI) are not appropriate for use in the Middle East culture. The aim of this study was to explore barriers against seeking help for UI and introducing a questionnaire that assess these barriers among those women. This is important before proceeding to any educational programs or having interval clinical audits to help incontinent women.

**Methods:**

*1- Screening for UI*. Women - aged 20 years and older, attending the outpatient Urology and Gynaecology clinics were invited to participate and interviewed by a research nurse. The UDI-6 was administered to assess the presence and type of UI. Women with UI as their chief complaint were excluded. *2- Interviewing study subjects for possible barriers*. Subjects who had UI - as determined by the UDI-6-were first asked an open question "what prevented you from seeking medical consultation for urine leakage?"." They were then asked the proposed questions to assess possible barriers. We developed a preliminary questionnaire based on a review of reasons for not seeking incontinence care from the literature and the response of UI sufferers to the open question in this study. The questionnaire was modified many times to reach this final form. 3- *Pilot Study to assess characteristics of the questionnaire*. Validity and reliability of the final version of the questionnaire were assessed in a small pilot study including 36 women who completed questionnaire at initial visit and again after 2 weeks.

**Results:**

Of the 1231 subjects who agreed to participate in the study, 348 reported having UI. About 80% of incontinent women have never sought medical advice. Factors significantly associated with seeking help were husband encouragement, prayer affection and having severe UI. Common barriers were embarrassment and assuming UI as a normal part of aging. A pilot study included 36 women to assess the psychometric properties of the questionnaire after modifying it. The number of missing or not interpretable responses per item ranged from 2.2% to 8.7%. Internal consistency of the items was good. The test-retest reliability of individual items of the questionnaire was variable, with weighted kappa statistics ranging from 0.32 to 0.94 (median, 0.76, p 0.000).

**Conclusions:**

Preliminary data on our proposed questionnaire show that it is an easy to administer, stable and suits the Middle Eastern culture.

## Background

Although urinary incontinence (UI) is a common debilitating and stigmatizing condition with substantial impact on quality of life (QoL), relatively few sufferers seek medical help[1]. The clinical and epidemiological criteria of UI are different among different cultures and ethnic groups. With reference to QoL issues, most affected women with UI in the Middle East have different QoL concerns including disruption of their prayer schedule and interference with sexual activity[2]. Consequently, it is expected that those women may have different and more complex barriers than their counterparts from other cultures. The Middle East-culture is a male-dominated one with conservative traditions and attitudes that may lead to unenthusiastic atmosphere toward UI[3]. Those women are underpowered minority in their societies and not expect to visit medical care except in emergencies "crisis oriented"[4]. In a previous study, [1] we found a marked discrepancy between a relatively high overall prevalence of UI among Egyptian women (55%) and a low consultation rate (4%); however, we did not go in depth to explore barriers that leaded to this low consultation rate. Such barriers have been studied for women living in Western and Industrialized countries by introducing an objective and psychometrically valid questionnaire[5]. However, this questionnaire is not suitable to be extrapolated to the Middle Eastern-women. Such a standardized tool is important for having clinical audits to asses the degree of progress and success of any educational program pertaining to UI.

The aim of this study was to explore the barriers against seeking help for UI among Egyptian women as a model of the Middle East women. We proposed a questionnaire which considered the social characteristics of this culture and introduced it for research and practical use. We then tested the psychometric properties of this questionnaire.

## Methods

### 1. Screening for UI

All women - aged 20 years and older, attending the outpatient clinics of Urology and Gynaecology departments of the University hospital (a tertiary referral centre), between May 2006 through December 2008, for whatever the indication - were invited to participate. Women with UI as their chief complaint were excluded from the study as those patients were actually seeking professional advice for incontinence. The study was approved by the ethical committee of the University. After informed consents were obtained from those who agreed to participate, study participants were interviewed anonymously in a private room by a trained research nurse. Data were collected by structured interview using the validated Urogenital Distress Inventory - short form (UDI-6) to assess the existence and type of UI[2].

### 2. Interviewing study subjects for possible barriers and modifying the questionnaire

Subjects with UI were asked questions inquiring about demographic data, obstetric, gynaecological and urological histories, including coital incontinence. Participants were first asked an open question to allow them to express their views in their own words: "*What prevented you from seeking medical consultation for urine leakage?*" Then they were asked the proposed questionnaire (V1) to assess possible barriers: "*To what extent does the following prevent you from seeking care for urine leakage?*". The response is a 3-Likert category ranging from "not at all (0)", "to some extent (1)" and "to great extent (2)". We developed this questionnaire based on a review of reasons for not seeking incontinence care from the literature [1,6] and the response of UI sufferers to the open question in this study. The research nurse provided nondirective assistance to those patients. Through individual patient interviews it was necessary to add and modify some items to get the second version of the questionnaire (V2). The final step consisted of testing the questionnaire on patients to determine whether it is acceptable, understandable in the way it is supposed to, and whether the language used is simple and appropriate. The questionnaire (V2) was administered to 6 women suffering from UI by in-person interview. During this interview, patients were asked if they had any difficulty understanding the questionnaire, and the patient's interpretation of all items was checked. Repeated modifications have been performed to reach the final version (V3) of the questionnaire. The questionnaire has been produced in 2 versions: Arabic and English for the non-Arabic speaking cultures.

### 3. Pilot Study to assess final version of the questionnaire

**Figure 1:**
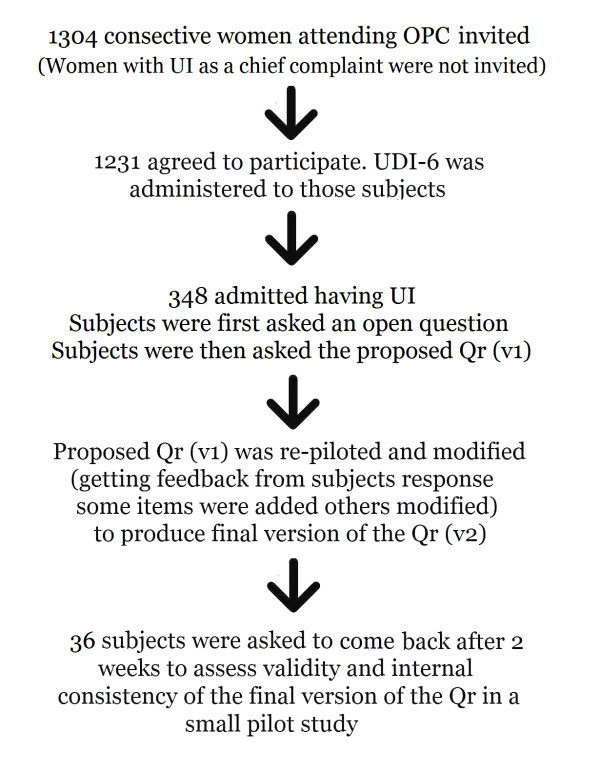
**Flow chart of study subjects**. OPC: outpatient Urology & Gynaecology clinics, Qr = Questionnaire, UDI-6 = Urogenital distress inventory, UI = Urinary Incontinence. The open question « what prevented you from seeking medical consultation for urine leakage?".

The final version of the questionnaire (V3) was administered to 36 women twice; 2 weeks apart to evaluate individual item performance (internal consistency) and test-retest reliability. Eligible participants were women suffering UI who had never sought medical consultation for their incontinence. Figure 1 shows the flow chart of study subjects. Individual items were evaluated by examination of patterns of response options and missing or not interpretable responses. Internal consistency among sets of items was evaluated with item-total correlations and the Cronbach α coefficient. Test-retest reliability of individual items was evaluated with weighted kappa statistics. Items that did not meet the criteria were revised.

### Statistical Analysis

The data analyzed came from a consecutive sample from the outpatient clinics, and no power calculations were performed in advance. The responses of study participants were recorded on structured survey forms and then entered into a computer database. The chi-squared test was used to compare potential factors between women who sought and those who did not seek consultation. A p-value less than 0.05 was considered to be statistically significant. In addition, a multivariate logistic regression analysis was used to consider the effects of all potential factors simultaneously. All potential factors were included in these regression models. SPSS software version 16.0 was used for the data analysis.

## Results

### 1. Screening for UI

A total of 1304 consecutive women were invited to participate in our study; 1231 agreed to participate, while 73 did not. Reasons for declining were not documented. The characteristics of study subjects are listed in Table 1. While 697 subjects (56.6%) came from the Gynaecology, 534 (43.3%) came from Urology clinic.

**Table 1:** Characteristics of study subjects

	n = 348 No. (%)
**Age in years (mean, range)**	40 (± 11.35)
20 - 29	70 (20%)
30 - 39	86 (24.7%)
40 - 49	115 (33%)
50 - 59	52 (14.9%)
≥ 60	25 (7.2%)

Educational background*:	
Illiterate	257 (73.9%)
Read and write	9 (2.6%)
Primary school level	12 (3.4%)
Prep school level	10 (2.9%)
Secondary school level	53 15.2%)
University level	7 (2%)

**Residence:**	
• Rural	234 (67.2%)
• Semi-urban	20 (5.7%)
• Urban	94 (27.0%)

**Religion:**	
• Muslim	335 (96.3%)
• Christian	13 (3.7%)

**Menstrual status:**	
• Premenopausal	278 (79.9%)
• Postmenopausal	70 (20.1%)

**Parity:**	4.63 (± 3.2)
• Nullipara	41 (11.8%)
• 1 - 3	95 (27.3%)
• > 3	212 (60.9%)

### 2. Interviewing study subjects for possible barriers

Univariate analysis of the potential factors associated with seeking consultation is listed in Table 2. Surprisingly, the educational levels of the woman or her husband were not significant promoting factors while husband encourage, prayer affection and occurrence of coital as well as severe UI were. On multivariate logistic regression analysis (Table 3), the strongest promoting factor was prayer affection. Barriers against seeking consultation for UI were embarrassment (67.2%), assuming UI a normal part of the aging or after giving birth of multiple children (46.7%), choice of self-treatment (39.2%), low expectations from medical care (38.2%), thinking UI may resolve spontaneously (15.8%), and thinking that treatment would be costly (12.6%). About 8.8% of incontinent women reported that their husband encouraged them to seek consultation, 77.3% stated that their husband did not find it a good idea, while 13.9% of women mentioned that their husbands were neutral about this idea. As regard further management, 29.6% of incontinent women reported that they can tolerate incontinence, 66.7% mentioned that they need conservative treatment for their incontinence, while only 3.8% mentioned they might proceed to do surgery if recommended. While 13% of incontinent women reported that their complaints were taken seriously by their primary health care provider; 87% mentioned that their physicians were reluctant to give them any further management. For those who were given treatment by the family doctor, 37.5% mentioned that their condition improved, 54.2% no change while 8.3% mentioned that their condition was worsened.

**Table 2:** Univariate analysis of factors promoting women to seek help for incontinence

Variable	Sought medical advise (n = 70)	p value
**Age**		
20-29	13 (18.6%)	0.861
30-39	16 (22.9%)	
40-49	22 (31.4%)	
50-59	13 (18.6%)	
≥ 60	6 (8.6%)	

**Parity**		
Nullipara	9 (12.9%)	0.643
1 - 3	16 (22.9%)	
> 3	45 (64.3%)	

**Religion**		
Muslim	68 (97.1%)	0.495
Christian	2 (2.9%)	

**Level of education**		
Illiterate	54 (77.1%)	0.904
Elementary (basic)	4 (5.7%)	
Secondary	11 (15.7%)	
Higher	1 (1.4%)	

**Residency**		0.457
Rural	52 (74.3%)	
Urban	18 (25.7%)	

**Type of incontinence**		0.000
Stress	31 (44.3%)	
Urge	8 (11.4%)	
Mixed	31 (44.3%)	

**Severe incontinence**	28 (40%)	0.000

**Affect prayer**	66 (94.3%)	0.000

**Coital incontinence**	26 (37.1%)	0.001

**Husband Encouragement**	16 (22.9%)	0.000

**Husband Educated**	21 (30%)	0.536

**Table 3:** Multivariate logistic regression analysis of the factors promoting Egyptian women suffering urinary incontinence to seek medical consultation

	OR	95.0% C.I. for EXP(B)		p value
			
		Lower	Upper	
**Age > 50 years**	.713	.083	6.098	.757
**Higher education level of the woman**	.266	.019	3.817	.330
**Urban Residence**	1.463	.706	3.030	.306
**Higher education level of the husband**	1.538	.619	3.824	.354
**Husband encouragement**	4.356	1.587	11.955	.004
**Postmenopausal Status**	1.114	.112	11.062	.927
**Multiparity (>3)**	1.477	.668	3.264	.335
**Prayer affection**	4.084	2.132	7.821	.000
**Stress Incontinence**	2.413	1.623	6.421	.002
**Coital incontinence**	1.042	.433	2.509	.927
**Severe incontinence**	2.695	1.378	5.271	.004

Odds ratio and confidence intervals are derived from a multivariate logistic model including all potential factors that might promote women to seek consultation, with the outcome as to seek medical consultation vs. never sought consultation.

### 3. Pilot testing

It included 36 subjects. The number of missing or not interpretable responses per item ranged from 2.2% to 8.7%. Internal consistency of items was good. The test-retest reliability of individual items of the questionnaire was variable, with weighted kappa statistics ranging from 0.32 to 0.94 (median, 0.76, p 0.000).

## Discussion

Although UI is a prevalent condition and occurs among relatively younger Egyptian women, few women, however, rarely seek medical help because of many barriers[1]. Only 4% of sufferers have sought medical advice compared to a relatively higher consultation rate in a European survey (31%)[7]. It is common that these women continue to live silently with incontinence[8]. Embarrassment and lack of awareness towards symptoms and availability of treatment options have been identified as barriers to help-seeking. UI is a very sensitive issue that some women find it shameful to discuss especially those with those with poor educational background. Middle East culture is a male-dominated society and where religion plays an important role in the society[9]. This is probably due to imbalance of the family power in these cultures created by having women not contributing in the financial income of the family. Other women perceive UI as an aging phenomenon rather than a pathological condition caused by childbirth or menopause. Symptoms are sometimes not felt to be serious enough, and the prioritizing of help-seeking for more serious conditions. Factors that strongly promoted women to seek consultation in our study were husband encouragement, followed by prayer affection for Muslims, and the severity of incontinence. Factors that promoted women in US were different, including symptom duration >3 years, having a history of a noticeable accident, worse QoL scores, not being embarrassed to talk with a physician about urinary symptoms, talking with others about UI, and keeping regular appointments for routine/preventive care[10].

Women whose primary complaint was UI were excluded from the study. One may argue that this may undermine the research hypothesis because "primarily" incontinent women are expected to be the target sample even if they were seeking hospital care. We were looking for women with UI who had barriers that prevent them to seek primary consultation for their incontinence. Although our study subjects were already in a hospital for problems other than incontinence, they could not cross the barriers to seek help for incontinence.

Heit et al.[5] established the psychometric properties of the Barriers to Incontinence Care Seeking questionnaire that contains 13 items and introduced it to research practice. However, this instrument is not suitable to assess barriers against UI seeking care among Egyptian and probably Middle Eastern women in its current form. For example, the availability of free health care in Governmental and University hospitals in Egypt tends to balance the traditional item barriers to UI care seeking in women found in their questionnaire. It was necessary to develop a questionnaire to assess barriers that considered societal characteristics of the Middle East culture (Additional file 1).

Another aspect of the problem comes from primary health care providers. Routine medical assessment of most of our study subjects by their physician did not include an inquiry about bladder symptoms. Even those who reported their incontinence to their doctors were disappointed. A relatively poor rates of accurate diagnosis by physicians were observed. Primary Health care providers should be taught how to screen these cases, treat simple cases and refer complex cases.

This work has implications for policy and practice and stresses the importance of the provision of health promotion information. The proposed questionnaire could be a good model for interval clinical audits to asses the degree of progress and success of the educational programs to educate not only women, but the whole society. Raising awareness should stem from the media. This investigatory tool hopefully may encourage health care professionals to assess patient attributions of their condition and treatments in order to avoid health behaviour that is informed by a poor knowledge base, and also to identify patient needs from the consultation, which may be information oriented rather than treatment oriented.

## Conclusions

Barriers that prevent Middle Eastern women from seeking medical consultation for UI are different from those of women in other communities. Most common barriers include the misconceptions about the causes of and availability of treatment options for UI and embarrassment. Our proposed questionnaire to assess these barriers has good internal consistency and test-retest reliability.

## Competing interests

The authors declare that they have no competing interests.

## Authors' contributions

**AE: **designed the study, collected the data on spread sheet, performed the statistical analysis and drafted the manuscript. **OS: **participated in designing the study, shared in collecting the data on spread sheet, and reviewed the manuscript

All authors read and approved the final manuscript.

## Pre-publication history

The pre-publication history for this paper can be accessed here:

http://www.biomedcentral.com/1472-6874/10/3/prepub

## Supplementary Material

Additional file 1 **Measuring the Barriers against Seeking Consultation for Urinary Incontinence among Middle Eastern Women**. the article explores the barriers that prevent Middle Eastern women from seeking medical consultation for urinary incontinence. Click here for file
