# Supplementary material for: Measuring the barriers against seeking consultation for urinary incontinence among Middle Eastern women
Source: BMC Womens Health. 2010 Jan 27;10:3. doi: 10.1186/1472-6874-10-3 (PMC2835642; doi:10.1186/1472-6874-10-3)
Supplement: Additional file 1 — Measuring the Barriers against Seeking Consultation for Urinary Incontinence among Middle Eastern Women. the article explores the barriers that prevent Middle Eastern women from seeking medical consultation for urinary incontinence. [file 1472-6874-10-3-S1.DOC]

**Appendix 1: Barriers to seek medical consultation for Incontinent Middle Eastern Women Questionnaire**

To what extent does the following prevent you from seeking care for urine leakage, please respond as many answer as suits your condition. Please rate your response as either: **Not at All**, **To Some Extent** or To a **Great Extent**

1. Embarrassed to discuss my problem with any health care provider
2. There is no need for medical help because incontinence is a normal consequence of aging
3. I prefer to discuss the problem with relatives or friends
4. Urinary incontinence may resolve spontaneously
5. I have low expectations from medical consultation
6. The health care provider will not be interested in my problem
7. I think the financial cost to solve my problem will be too high
